# Overexpression of miR-146a Might Regulate Polarization Transitions of BV-2 Cells Induced by High Glucose and Glucose Fluctuations

**DOI:** 10.3389/fendo.2019.00719

**Published:** 2019-10-22

**Authors:** Yinqiong Huang, Zhenling Liao, Xiahong Lin, Xiaohong Wu, Xiaoyu Chen, Xuefeng Bai, Yong Zhuang, Yingxia Yang, Jinying Zhang

**Affiliations:** ^1^Department of Endocrinology, The Second Affiliated Hospital of Fujian Medical University, Quanzhou, China; ^2^Department of Endocrinology, The First People's Hospital of Shaoguan City, Shaoguan, China; ^3^Department of Neurology, The Second Affiliated Hospital of Fujian Medical University, Quanzhou, China

**Keywords:** microglia, polarization transitions, miR-146a, neuroinflammation, glucose fluctuation

## Abstract

Microglia are critical in neuroinflammation. M1/M2 polarization transitions of microglial phenotypes determine the states of neuroinflammation and are regulated by multiple pathways, including miRNAs and other epigenetic regulations. This study investigated the polarization transitions of microglia induced by high glucose and glucose fluctuations, and the role of miR-146a in regulating M1/M2 polarization transitions of microglia. BV-2 cells were cultured with 25 mmol/L glucose, 75 mmol/L glucose, and 25 mmol/L−75 mmol/L glucose fluctuation for 48 h. BV-2 cells overexpressing miR-146a were generated using a lentiviral vector. Quantitative real-time polymerase chain reaction (qRT-PCR) was used to measure mRNA expression of miR-146a, CD11b, iNOS, Arg-1, IRAK1, TRAF6, and NF-κB. Immunofluorescence was used to measure CD11b expression. Western blot was used to measure protein expression of CD11b, iNOS, and Arg-1. Compared with those in the 25 mmol/L glucose control group, expression of CD11b, iNOS, TNF-α, and IL-6 in the 75 mmol/L glucose or glucose fluctuation groups of cultured BV-2 cells were significantly increased, while Arg-1 and IL-10 was significantly decreased. These effects were reversed by overexpression of miR-146a. Furthermore, expression of IRAK1, TRAF6, and NF-κB was significantly increased in the high glucose and glucose fluctuation groups; this was reduced after miR-146a overexpression. In sum, high glucose and glucose fluctuations induced polarization transitions from M1 to M2 phenotype in BV-2 cells. Overexpression of miR-146a might protect BV-2 cells from high glucose and glucose fluctuation associated with M1/M2 polarization transitions by downregulating the expression of IRAK1, TRAF6, and NF-κB.

## Introduction

Diabetic encephalopathy (DE) is a chronic complication of diabetes mellitus (DM), which can cause cognitive decline, dementia, and mental disorders, which significantly affects the quality of life of diabetic patients (1). Since the concept of DE was proposed by Nieleson in 1965, our understanding of DE has deepened. Its pathogenesis involves various aspects, including neuroinflammation (2, 3), blood-brain barrier (BBB) (2), vascular factors (4), insulin resistance, insulin deficiency (5, 6), and oxidative stress (3, 6). However, controversy in the literature remains. Neuroinflammation plays an important role in the pathogenesis of DE (7). Neuroinflammation is a complex innate immune response of the central nervous system that inhibits infection; removes pathogens, cell debris, and misfolded proteins; and plays an important role in nerve repair and evolution (8, 9). However, the persistence or excessive activation of neuroinflammation can lead to various neurological diseases.

Microglia are critical nervous system-specific immune cells involved in the response to neuroinflammation. Microglial polarization states comprise M1 and M2 phenotypes. M1 polarized microglia have pro-inflammatory effects and phagocytic functions (10), mainly secreting pro-inflammatory factors such as inducible nitric oxide synthase (iNOS), tumor necrosis factor (TNF)-α, interleukin (IL)-6, and nitric oxide (NO) to further aggravate neuronal damage. M2 polarized microglia inhibit inflammatory responses, regulate neuroinflammation (7), nourish nerves, and promote nerve repair (8, 11), with overexpression of arginase-1 (Arg-1), IL-10, IL-4, and transforming growth factor (TGF)-β. Polarization transitions to M1 phenotype induced by high glucose and glucose fluctuations have been observed in microglia both *in vivo* and *in vitro*. The expression of iNOS in the hippocampus of diabetic mice was significantly increased by inflammatory factors induced by high glucose (11). Compared with that in the control group, the expression of iNOS in microglia was upregulated after glucose fluctuations. The expression of iNOS also significantly increased after high glucose induction (12). Activation of nuclear factor (NF)-κB and signal transducer and activator of transcription 1 (STAT1) may regulate M1 polarization of microglia, whereas STAT6 and STAT3 activation may regulate M2 polarization (13). Nevertheless, it remains unclear how hyperglycemia and glucose fluctuations regulate polarization transitions of microglia.

In recent years, studies have reported that miRNAs are involved in the regulation of microglia polarization. miR-29b and miR-125a induce polarization transitions to M1 phenotype by inhibiting the expression of TNF-α-inducible protein 3 (TNFAIP3) downstream of the NF-κB signaling pathway (14). miR-146a plays an important role in the regulation of inflammatory responses (15). Previous studies confirmed that miR-146a directly regulates IRAK1 and TRAF6, thereby regulating the pro-inflammatory factor NF-κB and expression of inflammatory factors (16).

The present study therefore aimed to evaluate the role of miR-146a on high glucose and glucose fluctuation-associated polarization transitions of microglia. We hypothesized that miR-146a was involved in the regulation of polarization transitions of microglia induced by hyperglycemia and glucose fluctuations.

## Materials and Methods

### Cell Culture

BV-2 cells were obtained from Chinese Academy of Sciences (CAS) Kunming Cell Bank (Kunming, China) and cultured in 25 mmol/L glucose Dulbecco's Modified Eagle Medium (DMEM) (Gibco, USA) supplemented with 10% fetal bovine serum (FBS) (Gibco, USA) and 1% penicillin-streptomycin (Hyclone, USA). Cells were maintained in a humidified atmosphere containing 5% CO_2_ at 37°C.

For glucose fluctuation BV-2 group culture, after cell attachment, the cell culture medium was replaced with 25 or 75 mmol/L glucose in DMEM every 6 h and cultured for 48 h. High glucose was the last fluctuation.

Stably transfected BV-2 cells overexpressing miR-146a were generated using the overexpression plasmid vector GV369 (Addgene). miR-146a was amplified using the following primers: forward primer, 5′-GAGGATCCCCGGGTACCGGTACAGGGCTGGCAGGATCTG-3; reverse primer, 5′- CACACATTCCACAGGCTAGCCCCACTCTCTCCACTCTTCAAG-3 (Shanghai Gene Company, Shanghai, China). The amplified sequences were inserted into GV369 by a recombinant method (Genechem Company, Shanghai, China) according to the manufacturer's instructions. The resultant plasmid was then transfected into 293T cells to construct a lentivirus overexpressing miR-146a.

BV-2 at 3 × 10^4^/well (six well plates) were seeded and cultured for 12 h. A volume of 2 mL enhanced infection solution containing Lv-mmu-miR-146a (Genechem Company, Shanghai, China) with the corresponding viral load and control virus expressing spontaneous green fluorescent protein (GFP) with puromycin acetyltransferase was incubated with cells. The load of viral particles was quantified and normalized before the addiction to the cells. At 12 h after infection, the medium was replaced with conventional culture medium. After 72 h, GFP expression was observed under a fluorescence microscope (Leica, Germany). Complete medium containing 2 μg/mL puromycin (Sigma, Aldrich, USA) was used to screen virus-infected cells, and 1 g/mL was used for further screening. Quantitative real-time polymerase chain reaction (qRT-PCR) was used to assess relative expression levels of miR-146a in the Lv-mmu-miR-146a infection group.

### Immunofluorescence Staining of CD11b

Cells grown on glass coverslips were fixed with 4% paraformaldehyde, 0.1% Triton X-100, permeabilized, blocked with 1% bovine serum albumin (BSA), and incubated with anti-CD11b antibody (rat monoclonal antibody; Abcam, USA; 1: 500) at 4°C overnight. After washes with 1 × phosphate-buffered saline (PBS), cells were incubated with the corresponding secondary antibody conjugated with Alexa Fluor® 647 (donkey polyclonal secondary antibody to rat IgG, H&L; Abcam, USA; 1:100) in the dark for 1 h and analyzed using an inverted fluorescence microscope (Leica, Germany). 4′,6-diamidino-2-phenylindole (DAPI) was used to label cell nuclei. To assess BV-2 cell activation. ImageJ was used to calculate the expression of CD11b fluorescence intensity per scaffold. At least five slides were examined per treatment group for each experiment. A comparison of the fluorescence intensity of CD11b between the indicated groups was performed.

### Quantitative RT-PCR (qRT-PCR)

Total RNA was extracted with TRIzol (RNAiso Plus) (Takara, Japan). RNA was reversed transcribed into cDNA using the twostep method with PrimeScript™ RT reagent Kit with gDNA Eraser (Takara, Japan). Genomic DNA of miRNA was removed using Recombinant DNase I (RNase-free) (Takara, Japan). miRNA was reversed transcribed into cDNA using the Mir-X® miRNA FirstStrand Synthesis and SYBR® qRT-PCR (Takara, Japan). mRNA qRT-PCR was performed with the TB Green™ Premix Ex Taq™ (Tli RNaseH Plus) (Takara, Japan). miRNA qRT-PCR was performed with Mir-X™ miRNA FirstStrand Synthesis and SYBR® qRT-PCR (Takara, Japan). The primers used are shown in Table 1.

**Table 1:** Primers of qRT-PCR.

**Gene**		**Primers sequence**
CD11b	Forward	5′ GAGCATCAATAGCCAGCCTCAGTG 3′
	Reverse	5′ CCAACAGCCAGGTCCATCAAGC 3′
iNOS	Forward	5′ GTTTACCATGAGGCTGAAATCC 3′
	Reverse	5′ CCTCTTGTCTTTGACCCAGTAG 3′
Arg-1	Forward	5′ CATATCTGCCAAAGACATCGTG 3′
	Reverse	5′ GACATCAAAGCTCAGGTGAATC 3′
β-actin	Forward	5′ CTACCTCATGAAGATCCTGACC 3′
	Reverse	5′ CACAGCTTCTCTTTGATGTCAC 3′
NF-κB	Forward	5′ CAAAGACAAAGAGGAAGTGCAA 3′
	Reverse	5′ GATGGAATGTAATCCCACCGTA 3′
TRAF6	Forward	5′ GAAAATCAACTGTTTCCCGACA3′
	Reverse	5′ ACTTGATGATCCTCGAGATGTC 3′
IRAK1	Forward	5′ GTTATGTGCCGCTTCTACAAAG 3′
	Reverse	5′ GATGTGAACGAGGTCAGCTAC 3′
TNF-α	Forward	5′ ATGTCTCAGCCTCTTCTCATTC 3′
	Reverse	5′ GCTTGTCACTCGAATTTTGAGA 3′
IL-6	Forward	5′ CTCCCAACAGACCTGTCTATAC 3′
	Reverse	5′ CCATTGCACAACTCTTTTCTCA 3′
IL-10	Forward	5′ TGCTAACCGACTCCTTAATGCAGGAC 3′
	Reverse	5′ CCTTGATTTCTGGGCCATGCTTCTC 3′
U6	Forward	5′ CTCGCTTCGGCAGCACA 3′
	Reverse	5′ AACGCTTCACGAATTTGCGT 3′
miR-146a−5p	Forward	5′ CGCTGAGAACTGAATTCCATGGGTT 3′

*β-actin was used as mRNA and U6 as miRNA reference gene, with the 2^−ΔΔCt^ method used for quantitation. Triplicate experiments were performed and repeated at least 3 times*.

### Western Blot

Total cell proteins were extracted and equal amounts of protein (20 μg/sample) were separated by 10% SDS-PAGE and transferred onto PVDF membranes (Millipore, USA). After blocking with 5% skim milk at room temperature for 2 h, the membranes were incubated with primary antibodies targeting β-actin (mouse monoclonal antibody; Bioworld Technology, USA; 1:10,000), Cd11b (rabbit polyclonal antibody; Abcam, USA; 1:2,000), iNOS (rabbit polyclonal antibody; Abcam, USA; 1:600), and Arg-1 (rabbit polyclonal antibody; Abcam, USA; 1:1,000) overnight at 4°C. Then, membranes were incubated with secondary antibodies (anti-rabbit or anti-mouse IgG/HRP; CST; 1:5,000) for 2 h with shaking. After enhanced chemiluminescence (ECL) (Merck Millipore, Germany) reaction, protein bands were revealed on a Gel Imaging System (Syngene, USA). Gray values were analyzed with Image Lab software (Bio-Rad, USA) using β-actin as a loading control.

### Enzyme-Linked Immunosorbent Assay (ELISA)

The levels of TNF-α, IL-6, and IL-10 in cell culture supernatants were evaluated with an ELISA kit (ABclonal, China), according to the manufacturer's instructions.

### Statistical Analysis

SPSS 22.0 software (SPSS, USA) was used for data analysis. Data are expressed as mean ± standard deviation (SD). Group pairs were compared by *t*-tests. Normally distributed data were analyzed 292 using one-way analysis of variance (ANOVA), and non-normal 293 distributions were analyzed with the Kruskal–Wallis *H*-test. Statistical differences were 297 considered as significant if the *P* < 0.05.

## Results

### Effects of High Glucose and Glucose Fluctuation on miR-146a Expression in BV-2 Cells

**Figure 1:**
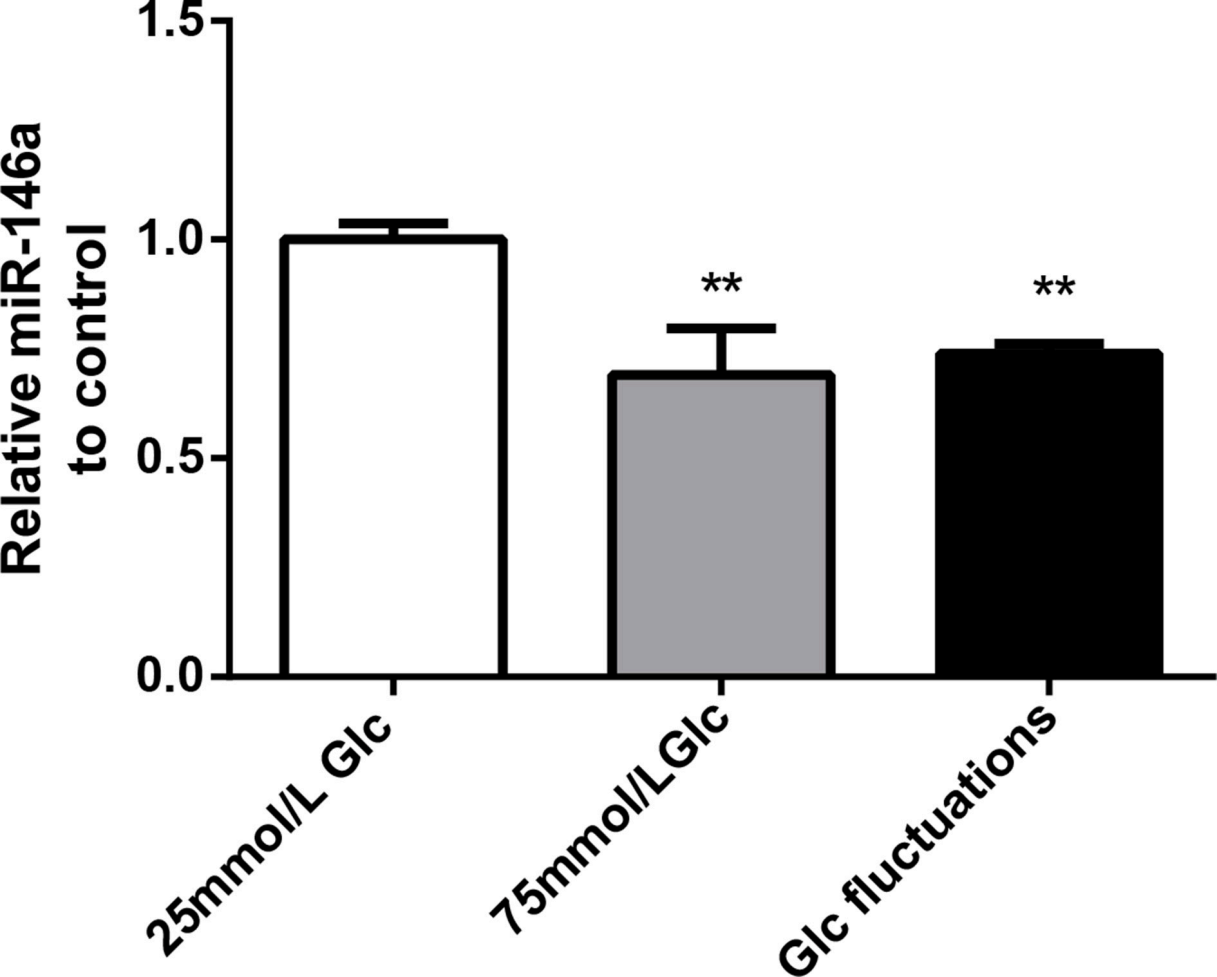
Effects of high glucose and glucose fluctuations on miR-146a levels in BV-2 cells. Expression levels of miR-146a, **p < 0.01 75 mmol/L Glc vs. control, **p < 0.01 Glc fluctuation vs. control.

Compared with that in the 25 mmol/L glucose group, the expression of miR-146a in the 75 mmol/L glucose group and glucose fluctuation group was significantly decreased (0.69 ± 0.107 vs. 1.00 ± 0.037, ^**^p = 0.001 and 0.74 ± 0.208 vs. 1.00 ± 0.037, ^**^p = 0.003, respectively) (Figure 1).

### miR-146a Overexpression in BV-2 Cells

**Figure 2:**
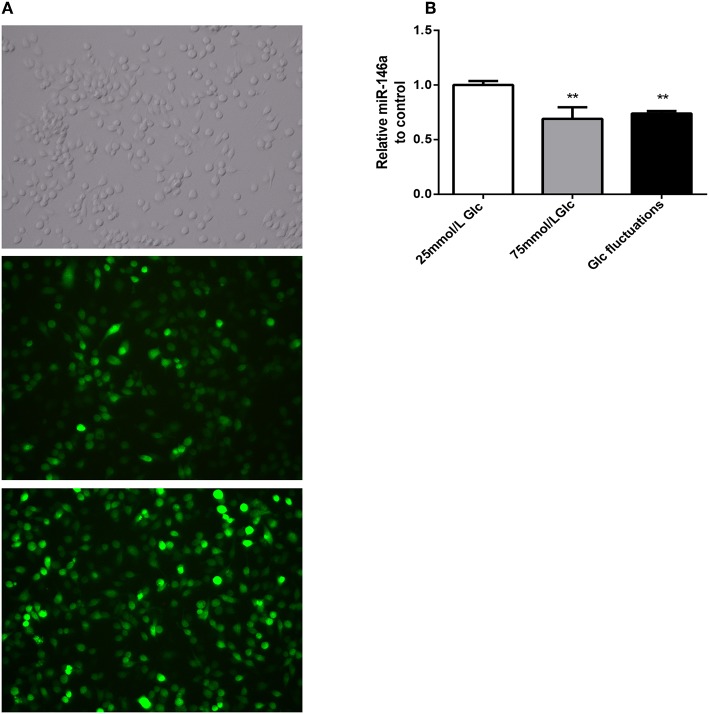
GFP expression and miR-146a expression levels after lentiviral infection in BV-2. **(A)** GFP expression after BV-2 transfection with over-expression of miR-146a lentivirus and control virus. **(B)** miR-146a expression levels before and after BV-2 transfection with over-expression of miR-146a lentivirus, ***p < 0.001 Lv-mmu-miR-146a group vs. control.

Green fluorescent protein was expressed by more than 80% of BV-2 cells 72 h after transfection with miR-146a lentivirus and control virus (Figure 2A). After stable transfection and puromycin screening, miR-146a levels in the Lv-mmu-miR-146a group were significantly higher than those of the control group (3.63 ± 0.208 vs. 1.00 ± 0.086, ^***^p < 0.001) (Figure 2B). These findings indicated that miR-146a overexpressing lentiviruses were successfully transfected into BV-2.

### Effects of miR-146a Overexpression on M1/M2 Polarization Transitions

#### Microglial Activation Marker CD11b

**Figure 3:**
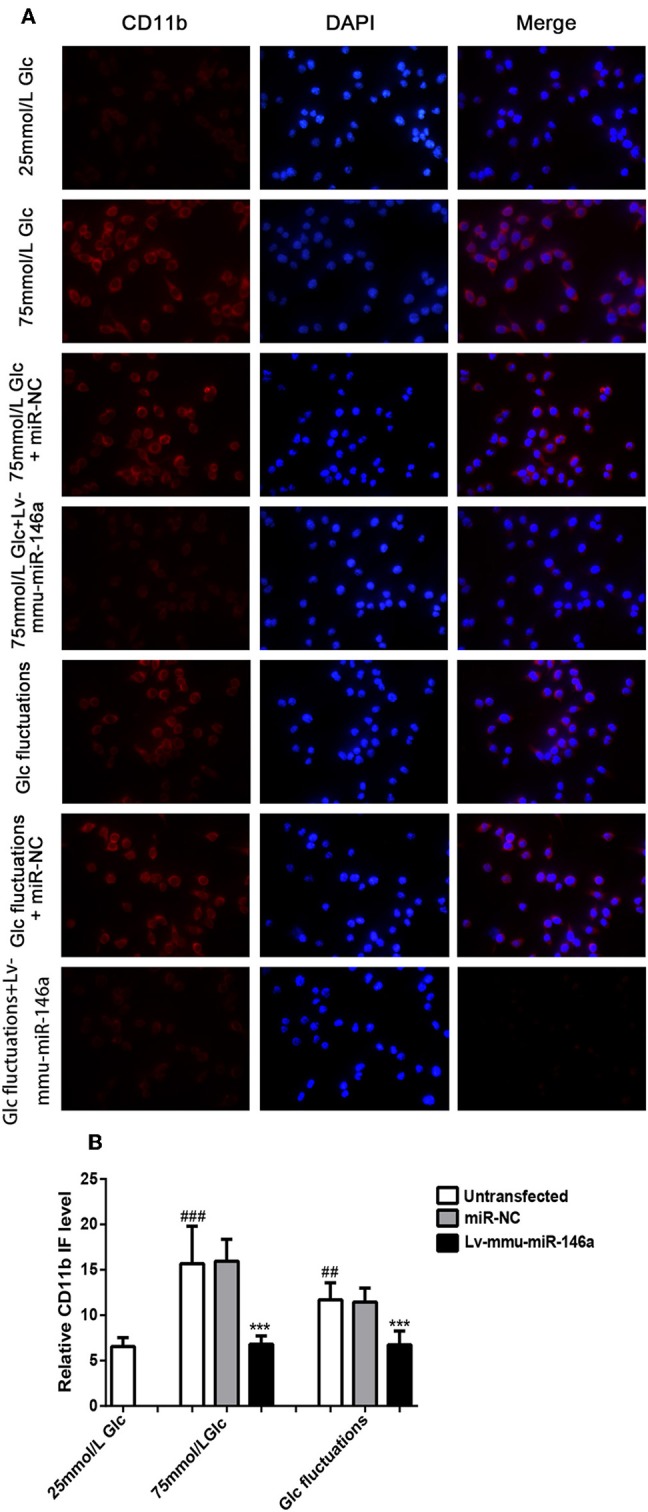
Immunofluorescence for CD11b in BV-2 cells overexpressing miR-146a induced by high glucose and glucose fluctuations for 48 h. **(A)** Immunofluorescence was used to measure the expression of CD11b protein in BV-2 cells overexpressing miR-146a induced by high glucose and glucose fluctuation. **(B)** Comparison of CD11b protein immunofluorescence intensity. ^*###*^*p* < 0.001, 75 mmol/L Glc vs. control; ^*##*^*p* < 0.01, Glc fluctuation group vs. control. ***p < 0.001, 75 mmol/L Glc+Lv-mmu-miR-146a group vs. 75 mmol/L Glc group, Glc fluctuation+Lv-mmu-miR-146a group vs. Glc fluctuation group.

Immunofluorescence analysis (Figure 3) indicated that the expression of the M1 phenotype polarization marker, CD11b, was significantly increased in the 75 mmol/L glucose (15.75 ± 3.98 vs. 6.47 ± 0.996, ^###^*p* < 0.001) and fluctuation group (11.5 ± 1.952 vs. 6.47 ± 0.996, ^##^*p* = 0.008) compared to that in the control group. The expression of CD11b was significantly decreased in the 75 mmol/L glucose+Lv-mmu-miR-146a group compared with that in the 75 mmol/L glucose group (6.74 ± 0.916 vs. 15.75 ± 3.98, ^***^p < 0.001). Similar results were observed in the glucose fluctuation+Lv-mmu-miR-146a group when compared with the glucose fluctuation group (6.76 ± 1.487 vs. 11.50 ± 1.952, ^***^p < 0.001).

**Figure 4:**
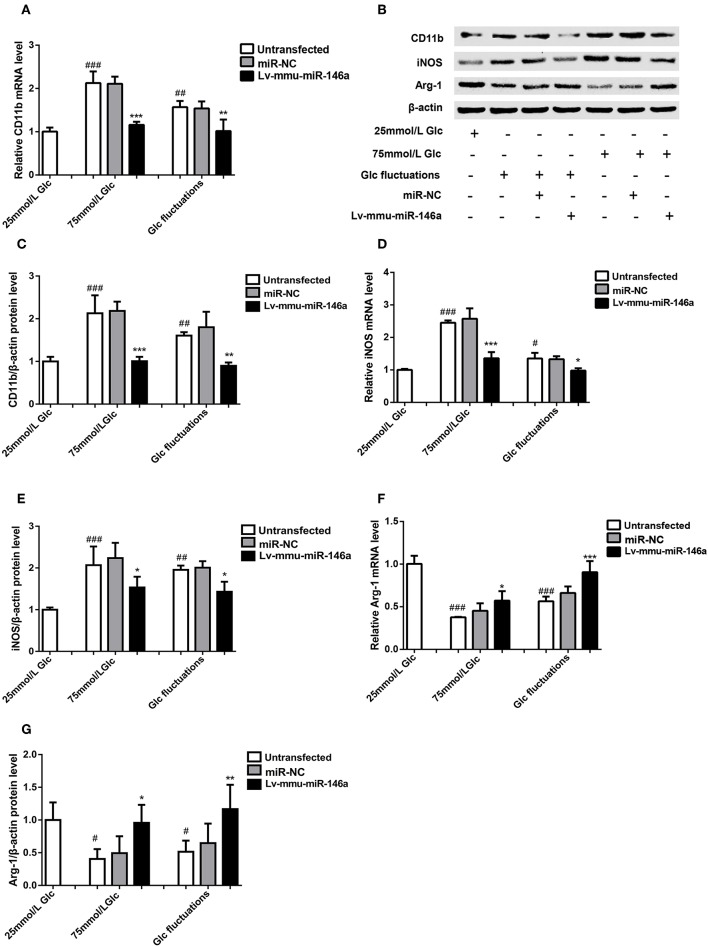
Effects of miR-146a on polarization transitions in BV-2 cells induced by high glucose and glucose fluctuation. **(A)** *CD11b* mRNA expression levels. ***p < 0.001, 75 mmol/L Glc+Lv-mmu-miR-146a group vs. 75 mmol/L Glc group; **p < 0.01, Glc fluctuation+Lv-mmu-miR-146a group vs. Glc fluctuation group; ^###^*p* < 0.001, 75 mmol/L Glc vs. control; ^##^*p* < 0.01, Glc fluctuation group vs. control. **(B)** Western blot for CD11b, iNOS, and Arg-1 protein detection. **(C)** Expression levels of CD11b protein. ***p < 0.001, 75 mmol/L Glc+Lv-mmu-miR-146a group vs. 75 mmol/L Glc group; **p < 0.01, Glc fluctuation+Lv-mmu-miR-146a group vs. Glc fluctuation group; ^##^*p* < 0.01, 75 mmol/L Glc vs. control; ^##^*p* < 0.01 Glc fluctuation group vs. control. **(D)** *iNOS* mRNA expression levels. ***p < 0.001, 75 mmol/L Glc+Lv-mmu-miR-146a group vs. 75 mmol/L Glc group; *p < 0.05 Glc fluctuation+Lv-mmu-miR-146a group vs. Glc fluctuation group, ^###^*p* < 0.001, 75 mmol/L Glc vs. control, ^#^*p* < 0.05, Glc fluctuation group vs. control. **(E)** Expression levels of iNOS protein. *p < 0.05 75 mmol/L Glc+Lv-mmu-miR-146a group vs. 75 mmol/L Glc group; *p < 0.05, Glc fluctuation+Lv-mmu-miR-146a group vs. Glc fluctuation group; ^###^*p* < 0.001, 75 mmol/L Glc vs. control; ^##^*p* < 0.01 Glc fluctuation group vs. control. **(F)** *Arg-1* mRNA expression levels. *p < 0.05 75 mmol/L, Glc+Lv-mmu-miR-146a group vs. 75 mmol/L Glc group; ***p < 0.001 Glc fluctuation+Lv-mmu-miR-146a group vs. Glc fluctuation group; ^###^*p* < 0.001, 75 mmol/L Glc vs. control; ^###^*p* < 0.001, Glc fluctuation group vs. control. **(G)** Expression levels of Arg-1 protein. *p < 0.05, 75 mmol/L Glc+Lv-mmu-miR-146a group vs. 75 mmol/L Glc group; **p < 0.01, Glc fluctuation+Lv-mmu-miR-146a group vs. Glc fluctuation group; ^#^*p* < 0.05, 75 mmol/L Glc vs. control; ^#^*p* < 0.05, Glc fluctuation group vs. control.

BV-2 cells were cultured in 25 mmol/L glucose, 75 mmol/L glucose, or glucose fluctuation groups for 24 h, and qRT-PCR was used to measure the expression of CD11b (Figure 4A). Compared with that in the 25 mmol/L glucose group, the expression of *CD11b* mRNA was significantly increased in the 75 mmol/L glucose group (2.13 ± 0.269 vs. 1.00 ± 0.093, ^###^*p* < 0.001) and glucose fluctuation group (1.57 ± 0.141 vs. 1.00 ± 0.093, ^##^*p* = 0.002). The expression of *CD11b* mRNA was significantly decreased in the 75 mmol/L glucose+Lv-mmu-miR-146a group compared with that in the 75 mmol/L glucose group (1.16 ± 0.074 vs. 2.13 ± 0.2691, ^***^p < 0.001). Similar results were observed in the glucose fluctuation+Lv-mmu-miR-146a group when compared with the glucose fluctuation group (1.015 ± 0.266 vs. 1.57 ± 0.141, ^**^p = 0.002).

Western blot (Figures 4B,C) revealed that the expression of CD11b protein was significantly increased in the 75 mmol/L glucose (2.13 ± 0.422 vs. 1.00 ± 0.110, ^##^*p* = 0.007) and fluctuation group (1.80 ± 0.360 vs. 1.00 ± 0.110, ^##^*p* = 0.003) compared with that in the control group. The expression of CD11b protein was significantly decreased in the 75 mmol/L glucose+Lv-mmu-miR-146a group compared with that in the 75 mmol/L glucose group (1.01 ± 0.102 vs. 2.13 ± 0.422, ^***^p < 0.001). Similar results were observed in the glucose fluctuation+Lv-mmu-miR-146a group when compared with the glucose fluctuation group (0.899 ± 0.077 vs. 1.80 ± 0.360, ^***^p < 0.001).

#### M1 Phenotype Polarization Marker iNOS

BV-2 cells were cultured in 25 mmol/L glucose, 75 mmol/L glucose, or glucose fluctuation groups for 24 h, and qRT-PCR was used to detect the expression of iNOS (Figure 4D). Compared with that in the 25 mmol/L glucose group, the expression of *iNOS* mRNA was significantly increased in the 75 mmol/L glucose group (2.45 ± 0.071 vs. 1.00 ± 0.030, ^###^*p* < 0.001) and glucose fluctuation group (1.35 ± 0.173 vs. 1.00 ± 0.030, ^#^*p* = 0.02). The expression of *iNOS* mRNA was significantly decreased in the 75 mmol/L glucose+Lv-mmu-miR-146a group compared with that in the 75 mmol/L glucose group (1.36 ± 0.188 vs. 2.45 ± 0.071, ^***^p < 0.001). Similar results were observed in the glucose fluctuation+Lv-mmu-miR-146a group when compared with the glucose fluctuation group (0.98 ± 0.072 vs. 1.35 ± 0.173, ^*^p = 0.014).

Western blot results (Figures 4B,E) revealed that the expression of iNOS protein was significantly increased in the 75 mmol/L glucose (2.07 ± 0.449 vs. 1.00 ± 0.055, ^#^*p* = 0.028) and fluctuation group (1.96 ± 0.104 vs. 1.00 ± 0.055, ^#^*p* = 0.03) compared with that in the control group. The expression of iNOS protein was significantly decreased in the 75 mmol/L glucose+Lv-mmu-miR-146a group compared with that in the 75 mmol/L glucose group (1.53 ± 0.257 vs. 2.07 ± 0.449, ^***^p < 0.001). Similar results were observed in the glucose fluctuation+Lv-mmu-miR-146a group when compared with the glucose fluctuation group (1.43 ± 0. 239 vs. 1.96 ± 0.104, ^**^p = 0.001).

#### M2 Phenotype Polarization Marker Arg-1

BV-2 cells were cultured in 25 mmol/L glucose, 75 mmol/L glucose, or glucose fluctuation groups for 24 h, and qRT-PCR was used to measure the expression of Arg-1 (Figure 4F). Compared to that in the 25 mmol/L glucose group, the expression of *Arg-1* mRNA was significantly decreased in the 75 mmol/L glucose group (0.38 ± 0.005 vs. 1.00 ± 0. 095, ^###^*p* < 0.001) and glucose fluctuation group (0.56 ± 0.055 vs. 1.00 ± 0.095, ^###^*p* < 0.001). The expression of *Arg-1* mRNA was significantly increased in the 75 mmol/L glucose+Lv-mmu-miR-146a group compared with that in the 75 mmol/L glucose group (0.57 ± 0.112 vs. 0.38 ± 0.005, ^*^p = 0.018). Similar results were observed in the glucose fluctuation+Lv-mmu-miR-146a group when compared with the glucose fluctuation group (0.91 ± 0.131 vs. 0.56 ± 0.055, ^***^p < 0.001).

Western blot results (Figures 4B,G) revealed that the expression of Arg-1 protein was significantly decreased in the 75 mmol/L glucose (0.41 ± 0.150 vs. 1.00 ± 0.269, ^#^*p* = 0.016) and fluctuation group (0.52 ± 0.170 vs. 1.00 ± 0.269, ^#^*p* = 0.042) compared with that in the control group. The expression of Arg-1 protein was significantly increased in the 75 mmol/L glucose+Lv-mmu-miR-146a group compared with that in the 75 mmol/L glucose group (0.96 ± 0.274 vs. 0.41 ± 0.150, ^*^p = 0.023). Similar results were observed in the glucose fluctuation+Lv-mmu-miR-146a group compared with that in the glucose fluctuation group (1.17 ± 0.373 vs. 0.52 ± 0.170, ^**^p = 0.009).

#### Inflammation Cytokines

##### TNF-α

**Figure 5:**
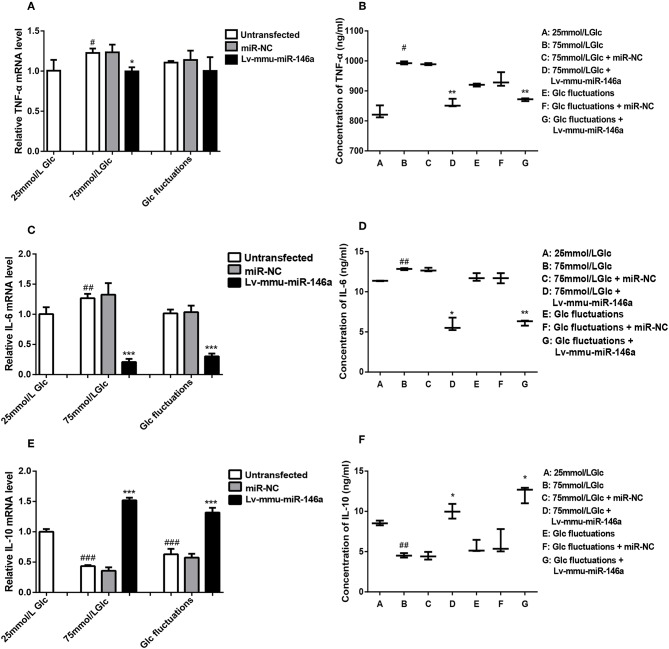
Effects of miR-146a on inflammatory cytokines expression in BV-2 cells induced by high glucose and glucose fluctuation. **(A)** TNF-α mRNA expression levels. ^#^*p* < 0.05, 75 mmol/L Glc vs. control; *p < 0.05, 75 mmol/L Glc+Lv-mmu-miR-146a group vs. 75 mmol/L Glc group. **(B)** ELISA results of TNF-α. ^#^*p* < 0.05, 75 mmol/L Glc vs. control; **p < 0.01, 75 mmol/L Glc+Lv-mmu-miR-146a group vs. 75 mmol/L Glc group; Glc fluctuation+Lv-mmu-miR-146a group vs. Glc fluctuation group. **(C)** IL-6 mRNA expression levels. ^##^*p* < 0.01, 75 mmol/L Glc vs. control; ***p < 0.001, 75 mmol/L Glc+Lv-mmu-miR-146a group vs. 75 mmol/L Glc group; ***p < 0.001, Glc fluctuation+Lv-mmu-miR-146a group vs. Glc fluctuation group. **(D)** ELISA results of IL-6. ^##^*p* < 0.01, 75 mmol/L Glc vs. control; *p < 0.05, 75 mmol/L Glc+Lv-mmu-miR-146a group vs. 75 mmol/L Glc group; **p < 0.01, Glc fluctuation+Lv-mmu-miR-146a group vs. Glc fluctuation group. **(E)** IL-10 mRNA expression levels. ^###^*p* < 0.001, 75 mmol/L Glc vs. control; ^###^*p* < 0.001, Glc fluctuation vs. control; ***p < 0.001, 75 mmol/L Glc+Lv-mmu-miR-146a group vs. 75 mmol/L Glc group. ***p < 0.001, Glc fluctuation+Lv-mmu-miR-146a group vs. Glc fluctuation group. **(F)** ELISA results of IL-10. ^##^*p* < 0.01, 75 mmol/L Glc vs. control; *p < 0.05, 75 mmol/L Glc+Lv-mmu-miR-146a group vs. 75 mmol/L Glc group; *p < 0.05, Glc fluctuation+Lv-mmu-miR-146a group vs. Glc fluctuation group.

BV-2 cells were cultured in 25 mmol/L glucose, 75 mmol/L glucose, or glucose fluctuation groups for 24 h, and qRT-PCR was used to measure the expression of TNF-α (Figure 5A). Compared to that in the 25 mmol/L glucose group, the expression of TNF-α mRNA was significantly increased in the 75 mmol/L glucose (1.23 ± 0.056 vs. 1.0 ± 0.134, ^#^*p* = 0.022). There was no significant difference between the fluctuation group and control group(1.11 ± 0.02 vs. 1.0 ± 0.134, *p* = 0.161). The expression of TNF-α mRNA was significantly decreased in the 75 mmol/L glucose+Lv-mmu-miR-146a group compared to the 75 mmol/L glucose group (1.0 ± 0.051 vs. 1.23 ± 0.056, ^*^p = 0.017). No significant difference between the glucose fluctuation+Lv-mmu-miR-146a group and the glucose fluctuation group (1.00 ± 0.172 vs. 1.11 ± 0.020, *p* = 0.368) was observed.

ELISA (Figure 5B) results showed that compared to that in the 25 mmol/L glucose group, the expression of TNF-α protein was significantly increased in the 75 mmol/L glucose (993.11 ± 5.06 vs. 828.09 ± 20.953, ^#^*p* = 0.02). No significant difference between the fluctuation group and control groups (919.70 ± 5.025 vs. 828.09 ± 20.953, *p* = 0.071) was observed. The expression of TNF-α protein was significantly decreased in the 75 mmol/L glucose+Lv-mmu-miR-146a group compared to the 75 mmol/L glucose group (857.74 ± 13.91 vs. 993.11 ± 5.06, ^**^p = 0.009), and significantly decreased in the glucose fluctuation+Lv-mmu-miR-146a group compared to the glucose fluctuation group (871.03 ± 4.649 vs. 919.7 ± 5.025, ^**^p = 0.003).

##### IL-6

BV-2 cells were cultured in 25 mmol/L glucose, 75 mmol/L glucose, or glucose fluctuation groups for 24 h, and qRT-PCR was used to measure the expression of IL-6 (Figure 5C). Compared to that in the 25 mmol/L glucose group, the expression of IL-6 mRNA was significantly increased in the 75 mmol/L glucose (1.27 ± 0.072 vs. 1.0 ± 0.114, ^##^*p* = 0.009). The expression of IL-6 mRNA was significantly decreased in the 75 mmol/L glucose+Lv-mmu-miR-146a group compared to the 75 mmol/L glucose group (0.21 ± 0.052 vs. 1.27 ± 0.072, ^*^p < 0.001), and similarly significantly decreased in the glucose fluctuation+Lv-mmu-miR-146a group compared to the glucose fluctuation group (0.30 ± 0.047 vs. 1.02 ± 0.064, ^***^P < 0.001).

ELISA (Figure 5D) results showed that compared to that in the 25 mmol/L glucose group, the expression of IL-6 protein was significantly increased in the 75 mmol/L glucose (12.830 ± 0.125 vs. 11.36 ± 0.032, ^##^*p* = 0.008 compared with control groups. No significant difference was observed between the fluctuation group and control groups (111.79 ± 0.483 vs. 11.36 ± 0.032, *p* = 0.826). The expression of IL-6 protein was significantly decreased in the 75 mmol/L glucose+Lv-mmu-miR-146a group compared to the 75 mmol/L glucose group (5.84 ± 0.828 vs. 12.830 ± 0.125, ^*^p = 0.02), and similarly significantly decreased in the glucose fluctuation+Lv-mmu-miR-146a group compared with the glucose fluctuation group (6.17 ± 0.343 vs 11.79 ± 0.483, ^**^p = 0.001).

##### IL-10

BV-2 cells were cultured in 25 mmol/L glucose, 75 mmol/L glucose, or glucose fluctuation groups for 24 h, and qRT-PCR was used to measure the expression of IL-10 (Figure 5E). Compared to that in the 25 mmol/L glucose group, the expression of IL-10 mRNA was significantly decreased in the 75 mmol/L glucose (0.434 ± 0.017 vs. 1.0 ± 0.045, ^###^*p* < 0.001)and in the fluctuation group (0.63 ± 0.089 vs. 1.0 ± 0.045, ^###^*p* < 0.001).The expression of IL-10 mRNA was significantly increased in the 75 mmol/L glucose+Lv-mmu-miR-146a group compared with the 75 mmol/L glucose group(1.52 ± 0.042 vs. 0.434 ± 0.017, ^***^p < 0.001), and similarly significantly increased in the glucose fluctuation+Lv-mmu-miR-146a group compared with the glucose fluctuation group (1.32 ± 0.078 vs. 0.63 ± 0.089, ^***^p < 0.001).

ELISA (Figure 5F) results showed that compared to that in the 25 mmol/L glucose group, the expression of IL-10 protein was significantly decreased in the 75 mmol/L glucose (4.53 ± 0.276 vs. 8.55 ± 0.296, ^##^*p* = 0.001). No significant difference was observed between the fluctuation group and control groups (5.56 ± 1.788 vs. 8.55 ± 0.296, *p* = 0.083). The expression of IL-10 protein was significantly increased in the 75 mmol/L glucose Lv-mmu-miR-146a group compared with the 75 mmol/L glucose group(10.0 ± 0.915 vs. 4.53 ± 0.276, ^*^p = 0.01), and similarly significantly increased in the glucose fluctuation Lv-mmu-miR-146a group compared with the glucose fluctuation group (12.21 ± 1.046 vs. 5.56 ± 0.788, ^*^p = 0.011).

### miR-146a Regulation of Polarization Transitions via IRAK1/TRAF6/NF-κB Signaling

**Figure 6:**
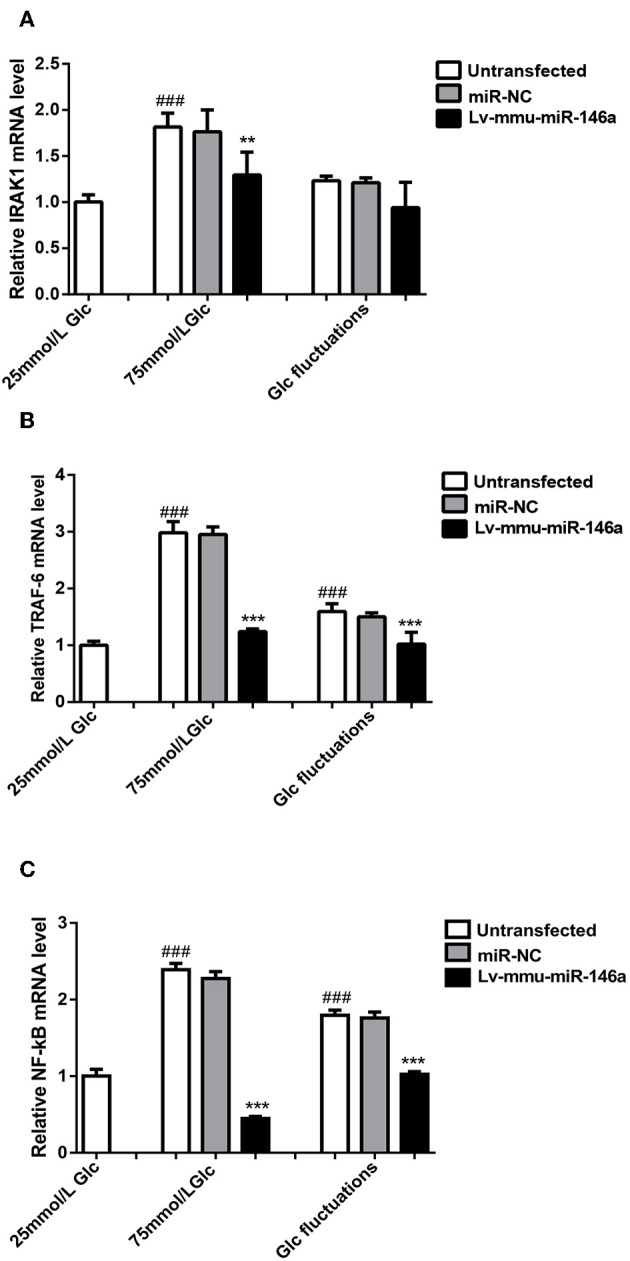
miR-146a regulation of polarization transitions via the IRAK1/TRAF6/NF-κB signaling pathway. **(A)** IRAK1 mRNA expression levels. ^*###*^*p* < 0.001, 75 mmol/L Glc vs. control; **p < 0.01, 75 mmol/L Glc+Lv-mmu-miR-146a group vs. 75 mmol/L Glc group. **(B)** TRAF6 mRNA expression levels. ^*###*^*p* < 0.001, 75 mmol/L Glc vs. control, Glc fluctuation group vs. control; ***p < 0.001, 75 mmol/L Glc+Lv-mmu-miR-146a group vs. 75 mmol/L Glc group, Glc fluctuation+Lv-mmu-miR-146a group vs. Glc fluctuation group. **(C)** NF-κB mRNA expression levels. ^*###*^*p* < 0.001, 75 mmol/L Glc vs. control, Glc fluctuation group vs. control; ***p < 0.001, 75 mmol/L Glc+Lv-mmu-miR-146a group vs. 75 mmol/L Glc group, Glc fluctuation+Lv-mmu-miR-146a group vs. Glc fluctuation group.

The expression of *IRAK1* mRNA (Figure 6A) was significantly increased in the 75 mmol/L glucose (1.82 ± 0.151 vs. 1.0 ± 0.079, ^###^*p* < 0.001) compared with that in the control group. No significant difference between the fluctuation group and control group was observed (1.23 ± 0.05 vs. 1.0 ± 0.079, *p* = 0.067). The expression of *IRAK1* mRNA was significantly decreased in the 75 mmol/L glucose+Lv-mmu-miR-146a group compared with that in the 75 mmol/L glucose group (1.3 ± 0.247 vs. 1.82 ± 0.151, ^**^p = 0.03). No significant difference between the glucose fluctuation+Lv-mmu-miR-146a group and glucose fluctuation group was observed (0.94 ± 0.275 vs. 1.23 ± 0.05, *p* = 0.14).

The expression of *TRAF6* mRNA (Figure 6B) was significantly increased in the 75 mmol/L glucose (2.98 ± 0.199 vs. 1.0 ± 0.071, ^###^*p* < 0.001) and fluctuation group (1.59 ± 0.137 vs. 1.0 ± 0.071, ^###^*p* < 0.001) compared with that in the control group. The expression of *TRAF6* mRNA was significantly decreased in the 75 mmol/L glucose+Lv-mmu-miR-146a group compared with that in the 75 mmol/L glucose group (1.24 ± 0.048 vs. 2.98 ± 0.199, ^***^p < 0.001), as well as in the glucose fluctuation+Lv-mmu-miR-146a group compared with that in the glucose fluctuation group (1.02 ± 0.207 vs. 1.59 ± 0.137, ^***^p < 0.001).

The expression of NF-κB mRNA (Figure 6C) was significantly increased in the 75 mmol/L glucose (2.39 ± 0.083 vs. 1.0 ± 0.088, ^###^*p* < 0.001) and fluctuation group (1.8 ± 0.07 vs. 1.0 ± 0.088, ^###^*p* < 0.001) compared with that in the control group. The expression of NF-κB mRNA was significantly decreased in the 75 mmol/L glucose+Lv-mmu-miR-146a group compared with that in the 75 mmol/L glucose group (0.6 ± 0.287 vs. 2.39 ± 0.083, ^***^p < 0.001), as well as in the glucose fluctuation+Lv-mmu-miR-146a group compared with that in the glucose fluctuation group (1.03 ± 0.036 vs. 1.8 ± 0.07, ^***^p < 0.001).

## Discussion

This study demonstrated that high glucose and glucose fluctuations induced polarization transitions to M1 phenotype in BV-2 cells. M1 phenotype parameters including CD11b and iNOS were significantly increased while the expression of M2 phenotype polarizing parameter Arg-1 was significantly decreased. These effects were reversed by overexpression of miR-146a. Furthermore, IRAK1, TRAF6, and NF-κB expression was significantly increased in the high glucose group and glucose fluctuation group. Similarly, these effects were reduced after miR-146a overexpression.

Neuroinflammation is a double-edged sword. Neuroinflammation plays an important role in suppressing infection, removing pathogens, clearing cell debris and misfolded proteins in the nervous system, and nerve repair. However, persistent neuroinflammation induces damage to the nervous system. The role of neuroinflammation depends primarily on the polarization transitions of M1/M2 phenotypes of microglia. Microglia are critical cells that mediate neuroinflammation and have different effects according to polarization phenotype. The M1-polarized phenotype secretes inflammatory cytokines and damages nerve cells, while the M2-polarized phenotype exerts protective effects on nerve cells. Most microglia in the central nervous system are in a relative “quiescent state.” Upon changes to the surrounding environment, microglia are activated, which induces M1/M2 polarization transitions. An *in vitro* study suggested that hyperglycemia induced polarization transitions to M1 polarization in microglia (17). In the cortex of diabetic mice, M1 phenotype polarization of microglia was increased, whereas M2 phenotype polarization of microglia was decreased. Similarly, microglia in the hypothalamus of streptozotocin-induced diabetic rats demonstrated M1 phenotype polarization transition (18). The deleterious effects of glucose fluctuations on diabetic macroangiopathy has been reported (19, 20). However, few studies on the effects of diabetic encephalopathy have been performed. An *in vitro* study indicated that glucose fluctuations significantly activated microglia (12). Nevertheless, more studies are required to confirm these findings.

In this study, we use 25 mmol/L glucose DMEM for BV-2 cell culture. We found that 25 mmol/L glucose didn't impact the level of miR-146a, while in the high glucose group, miR-146a expression was reduced. However, Chen et al. demonstrated that 25 mmol/L of glucose decreased the expression of miR-146a in human retinal microvascular endothelial cells (21).

We measured the microglial activation marker CD11b as well as M1 and M2 phenotype markers. We observed that both hyperglycemia and glucose fluctuations induced polarization transitions to M1 phenotype in microglia, leading to increased expression of pro-inflammatory factors including TNF-α and IL-6. Previous studies suggested that excessive M1 phenotype polarization induces inflammatory factor secretion in diabetic encephalopathy, leading to central nervous system damage in diabetes. The expression of iNOS was up-regulated in the hippocampus of diabetic mice and aggravated neuronal damage (22). Our study verified that high glucose and glucose fluctuations induced M1 phenotype polarization transitions in microglia *in vitro*, which is a possible mechanism underpinning diabetic encephalopathy.

Hyperglycemia and blood glucose fluctuations induce cell activation through multiple pathways (23). Of these, the TLR4/NF-κB signaling pathway is one of the main pathways involved. Dasu et al. reported that high glucose induced up-regulation of toll-like receptors (TLR; including TLR2 and TLR4) in human monocytic THP-1 cell lines and initiated intracellular myeloid differentiation factor 88 (MyD88)/IRAK-1/NF-κB signaling pathway, which induced inflammatory responses (24). In recent years, a growing number of studies has reported that epigenetic regulation is an important regulatory mechanism underscoring neuroinflammation, especially microRNAs (25–28). Different miRNAs are expressed depending on phenotype polarization of microglia; in turn, miRNA affect polarization transitions of microglia (29). For example, high expression of miR-155 in microglia induced M1 phenotype polarization and inhibited M2 phenotype polarization (30). miR-124 induced M2 phenotype polarization by targeting inhibition of M1 phenotype polarization to maintain the resting state of microglia and macrophages (31). miR-689, miR-124, and miR-155 are involved in M1 phenotype polarization. In contrast, miR-124, miR-711, and miR-145 are implicated in M2 phenotype polarization in lipopolysaccharide (LPS) or IL-4 stimulated primary mouse microglia based on miRNA expression profiling and bioinformatics analysis (29). To date, there have been no reports on miR-146a in the context of microglial phenotype polarization. Previous studies reported that miRNA-146a played an important role in the regulation of inflammation mainly through the NF-κB pathway. Taganov et al. reported that miR-146a regulated two key adapter molecules, TRAF-6 and IRAK1, which were downstream of the TLR signaling pathway, thereby regulating the activation of the pro-inflammatory factor NF-κB and negatively regulating the release of pro-inflammatory factors (16). Yang et al. reported that miRNA-146a negatively regulated mouse T-cell activation, and the regulatory mechanism also affected the expression of NF-κB by regulating the expression of TRAF-6 and IRAK1 (32). Studies have indicated that miR-146a inhibited M1 polarization and induced M2 polarization in mouse alveolar macrophages induced by LPS (33). Further, the M1 phenotype polarizing factors IL-6 and TNF-α were increased, while the M2 phenotype polarizing factor MGL-1 was decreased in alveolar macrophages by inhibiting the expression of miR-155 or miR-146a. miR-146a and miR-155 may be involved in the anti-inflammatory activity of macrophages and participate in the mechanisms underpinning M1 and M2 polarization transitions (34).

miR-146a induced M2 phenotype polarization and up-regulated tumor-associated macrophages expression to regulate breast cancer tumor growth (35). Our results showed that high glucose increased TNF-α and IL-6 expression while reduced IL-10 expression, which was reversed by overexpression of miR-146a. Similar results was observed in another study. Overexpression of miR-146a attenuated the inhibitory effects of NIFK-AS1 M2 phenotype polarization in macrophages, while the expression of IL-10 and Arg-1 were increased (36). Altered expression of miR-146a in the brains of patients with Alzheimer's disease (AD) underpinned inflammatory senile plaque lesions in AD brains (37, 38). miR-146a expression was decreased in DRG cells in hyperglycemia studies of diabetic peripheral neuropathy; cell survival rate was decreased, but this was attenuated by miR-146a mimics (39, 40). Rong et al. reported that the expression of miR-146a in plasma of patients with type 2 diabetes was significantly decreased (29). Our study revealed that the expression of miR-146a was significantly decreased in cell cultures by high glucose and glucose fluctuations, which partially verified that low levels of miR-146a expression were induced by hyperglycemia (41). Both *in vitro* and *in vivo* studies suggest that miR146a is involved in the regulation of neuroinflammation and plays an important role in various diseases such as diabetes and neurodegenerative diseases. Therefore, we speculated that miR-146a was involved in regulating the polarization transitions of microglia induced by high glucose and glucose fluctuations. Overexpression of miR-146a regulated polarization transitions induced by high glucose and glucose fluctuations promoting M1 to M2 phenotype polarization.

A negative feedback loop between miR-146a and NF-κB may be at play in this process. The expression of miR-146a was decreased in the hippocampus of diabetic rats, while increased expression of IRAK1, TRAF6, and NF-kB aggravated hippocampal inflammation and apoptosis (42). However, compared with that in the control group, the expression of miR-146a and NF-κB were increased, expression of TRAF6 was decreased, and expression of TNF-α, IL-6, and IL-1β were increased in the sciatic nerve of DM rats, indicating that loss of NF-κB/miR-146a in the negative feedback loop may underpin the pathogenesis of diabetic peripheral neuropathy (43). Further, miR-146a regulates the expression of target genes *IRAK1, TRAF6*, and NF-κB. Thus, the regulation may be involved in the NF-κB pathway. In this study, overexpression of miR-146a regulated M1/M2 polarization transitions by downregulating the expression of IRAK1, TRAF6, and NF-κB in microglia. This could be a regulatory mechanism in the response of microglial inflammation induced by high glucose and glucose fluctuation.

Central nervous system inflammation is a complex process involving various glial cells, macrophages, neuronal cells, and vascular endothelial cells. The results of this study were only observed *in vitro*, which precludes inferences on the neuroinflammatory effects of polarization transitions of microglia in diabetic encephalopathy. However, this study provides insight into the pathogenesis of diabetic encephalopathy. We observed that overexpression of miR-146a contributed to polarization transitions from M1 to M2 phenotype in microglia. Our results provide a potential strategy for the treatment of diabetic encephalopathy. Future studies should further investigate how miR-146a regulates polarization transitions in microglia induced by high glucose and glucose fluctuations.

## Data Availability Statement

All datasets generated for this study are included in the manuscript.

## Author Contributions

YH, ZL, and XL conceptualized and designed these studies, performed them, and wrote the manuscript. XW, XC, XB, YZ, YY, and JZ contributed through data analyses, data interpretation, and manuscript preparation. All authors contributed to manuscript revision and read and approved the submitted version.

### Conflict of Interest

The authors declare that the research was conducted in the absence of any commercial or financial relationships that could be construed as a potential conflict of interest.
